# Photonic structures in radiative cooling

**DOI:** 10.1038/s41377-023-01119-0

**Published:** 2023-06-01

**Authors:** Minjae Lee, Gwansik Kim, Yeongju Jung, Kyung Rok Pyun, Jinwoo Lee, Byung-Wook Kim, Seung Hwan Ko

**Affiliations:** 1 Applied Nano and Thermal Science Lab, Department of Mechanical Engineering, Seoul National University, 1 Gwanak-ro, Gwanak-gu, Seoul, 08826 South Korea; 2 Electronic Device Research Team, Hyundai Motor Group, 37, Cheoldobangmulgwan-ro, Uiwang-si, Gyeonggi-do 16082 South Korea; 3 E-drive Materials Research Team, Hyundai Motor Group, 37, Cheoldobangmulgwan-ro, Uiwang-si, Gyeonggi-do 16082 South Korea; 4 Department of Mechanical Robotics, and Energy Engineering, Dongguk University, 30 pildong-ro 1-gil, Jung-gu, Seoul, 04620 South Korea; 5 Department of Civil Engineering and Engineering Mechanics, Columbia University, New York, NY 10027 USA; 6 Institute of Advanced Machinery and Design (SNU-IAMD)/Institute of Engineering Research, Seoul National University, 1 Gwanak-ro, Gwanak-gu, Seoul, 08826 South Korea

**Keywords:** Applied optics, Optical materials and structures, Electronics, photonics and device physics

## Abstract

Radiative cooling is a passive cooling technology without any energy consumption, compared to conventional cooling technologies that require power sources and dump waste heat into the surroundings. For decades, many radiative cooling studies have been introduced but its applications are mostly restricted to nighttime use only. Recently, the emergence of photonic technologies to achieves daytime radiative cooling overcome the performance limitations. For example, broadband and selective emissions in mid-IR and high reflectance in the solar spectral range have already been demonstrated. This review article discusses the fundamentals of thermodynamic heat transfer that motivates radiative cooling. Several photonic structures such as multilayer, periodical, random; derived from nature, and associated design procedures were thoroughly discussed. Photonic integration with new functionality significantly enhances the efficiency of radiative cooling technologies such as colored, transparent, and switchable radiative cooling applications has been developed. The commercial applications such as reducing cooling loads in vehicles, increasing the power generation of solar cells, generating electricity, saving water, and personal thermal regulation are also summarized. Lastly, perspectives on radiative cooling and emerging issues with potential solution strategies are discussed.

## Introduction

By 2050, the demand for cooling energy of air conditioning is expected to rise significantly to 750% due to global warming as well as improvements in lifestyle in emerging economies^1^. The traditional cooling technologies accelerate global warming due to the use of refrigerants. This leads to the increased use of air conditioning, which consumes nonrenewable fossil energy for cooling and thus causes the Earth to become hotter^2,3^. More people are interested in increasing the effectiveness of current cooling systems and exploring new alternative cooling technologies by virtue of their increased knowledge of the energy situation and environmental concerns. Radiative cooling is a passive cooling technology that releases heat energy from a hot object to the largest cold object, the space^4^. Compared to conventional cooling technologies that require power sources and dump waste heat into the surroundings, radiative cooling is a heat dissipation through the space without any energy consumption^5–9^.

Radiative cooling is a thermal radiation process that carries heat energy. When a hot object and a cold object undergo radiative exchange, there is a net heat flow from the hot to the cold object^10,11^. Such a heat flow by thermal radiation leads to radiative cooling. Radiative cooling happens in our everyday life. For example, clear nights lead to cooler weather during autumn and winter. The Earth at ~300 K is hotter than the universe at 3 K^12^. Hence, the Earth emits the heat of thermal radiation to the universe through the atmosphere. Moreover, the Earth has a largely transparent atmospheric area in the mid-infrared wavelength range of 8–13 µm. This area, referred to as the atmospheric window, overlaps with the spectral peak of the thermal radiation due to the Earth’s temperature, and the heat escaping from the Earth’s surface to the universe via the atmospheric window causes a temperature drop. Consequently, the Earth’s heat radiations in the mid-infrared range through the atmospheric window to the cold space cause a rapid temperature drop at night. This nighttime radiative cooling in nature has been recognized and investigated systematically for many decades^13–16^.

**Fig. 1:**
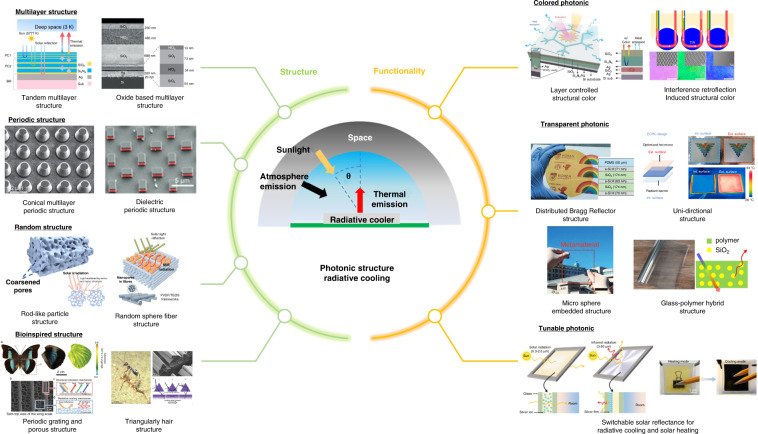
Outline for radiative cooling. Reproduced with permission^38^. Copyright 2019, American Chemical Society, Reproduced with permission^34^. Springer Nature¸ Reproduced with permission^65^. Copyright 2015, Wiley-VCH, Reproduced with permission^67^. Copyright 2017, Wiley-VCH, Reproduced with permission^78^. Copyright 2022, Elsevier, Reproduced with permission^73^. Copyright 2019, Wiley-VCH, Reproduced with permission^166^. Copyright 2021, Wiley-VCH, Reproduced with permission^103^, Copyright 2022, Royal society of chemistry, Reproduced with permission^99^. Copyright 2015, AAAS, Reproduced with permission^141^. Copyright 2018, Wiley-VCH, Reproduced with permission^144^. Copyright 2022, ACS, Reproduced with permission^166^. Copyright 2021, Wiley-VCH, Reproduced with permission^167^. Copyright 2022, Springer Nature, Reproduced with permission^168^. Copyright 2021, Wiley-VCH, Reproduced with permission^169^. Copyright 2021, KeAi, Reproduced with permission^201^. Copyright 2022, Cell press

Although radiative cooling occurs in nature, its performance must be controlled for practical applications. The nighttime radiative cooling cannot be used under the direct sun since the cooler absorbs the solar spectral heat from the sun during the day. Moreover, it is important to perform daytime radiative cooling under the direct sun since the cooling demands typically solar peak during the day. Therefore, simultaneous control of the absorptivity in the solar spectrum and the emissivity in the mid-infrared range becomes essential. Recently, nanophotonic structures designing thin multilayer and subwavelength grating structures have been demonstrated to control solar radiation and thermal radiation over specific wavelength^17–24^. This work proposes to review photonic radiative cooling from its fundamental principles to photonic development and its applications in various environments. Recent progress in multilayer, periodical, random, biomimetic, colored, transparent, and tunable photonic radiative cooling techniques are highlighted (Fig. 1). This work will demonstrate that photonic radiative cooling offers a potential route to energy conservation and more effective cooling load reduction in various practical applications.

This review article discusses the fundamentals of thermodynamic heat transfer that motivates radiative cooling in “Fundamentals of radiative cooling”. The photonics concept used in photonic radiative cooling is reviewed in “Photonic radiative cooling”. Furthermore, the radiative cooling technologies and their practical applications are discussed in “Application”.

## Fundamentals of radiative cooling

**Fig. 2:**
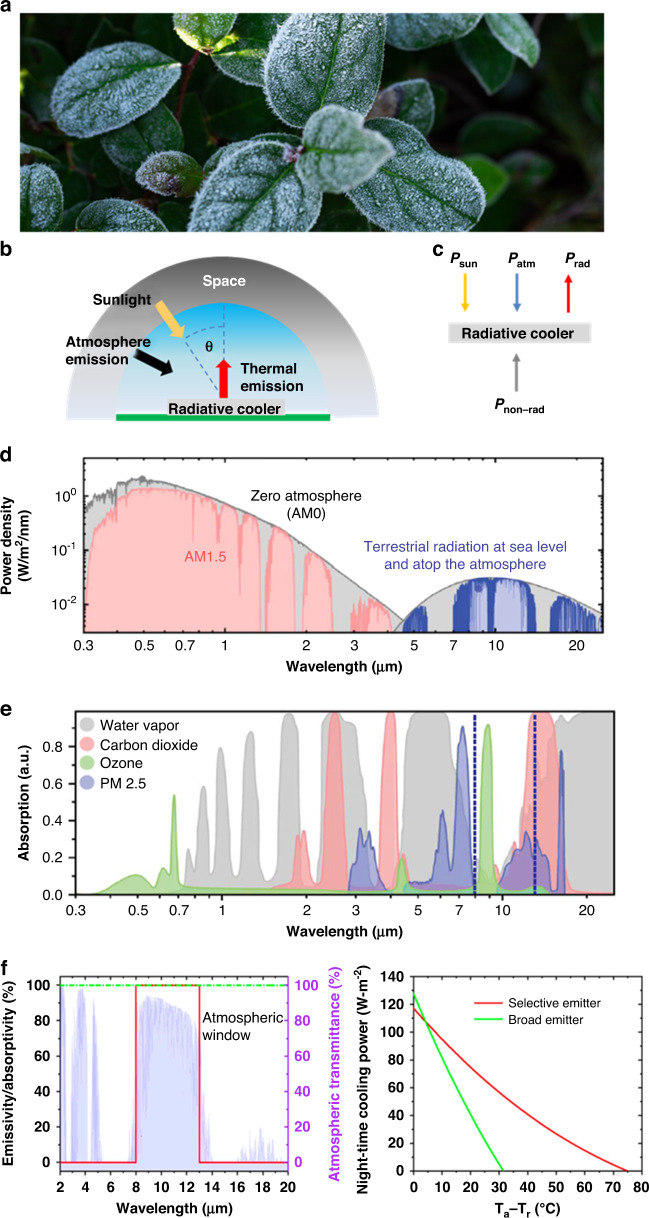
Fundamentals of radiative cooling. **a** Frozen iced on leaf in autumn. Reproduced with permission^25^. Copyright, Adobe Stock **b** Daytime radiative cooling heat exchange process. **c** Energy balance of radiative cooling **d** Solar spectra and terrestrial radiation spectra, respectively, at sea level and atop the atmosphere. **e** absorption spectra of gases in solar and mid-IR range. **d**, **e** Reproduced with permission^26^. Copyright 2018, AAAS. **f** Emissivity spectra of broadband and selective emitter. Reproduced with permission^30^. Copyright 2020, MDPI

Ice frozen on a leaf may be observed in early spring and late autumn (Fig. 2a)^25^, where the environmental temperature is above the frozen temperature, and more heat of solidification must be emitted for freezing. To cool down the hot object, it is located next to the cold object. Then heat exchange in thermodynamics occurs between two objects of different temperatures by emitting and absorbing the heat. A radiative transfer method carries the heat energy in electromagnetic waves. Since all objects above 0 K emit and absorb electromagnetic waves, the Earth, with a surface temperature near 300 K, also emits thermal radiation to space, at ~3 K, by radiative heat transfer. Looking at the radiative heat exchange in the Earth, the leaf facing the sky dissipates the heat through the atmospheric window ranging 8–13 µm to the space, the coldest reservoir, which can drop the surface temperature of the leaf below the air temperature, leading to radiative cooling.

Regarding the radiative cooling of the leaf under sunlight, according to the energy balance of thermodynamics, the leaf is considered a radiative cooler with an area *A* at a temperature *T* that emits thermal radiation to the space (Fig. 2b); it is affected by solar radiation, atmospheric thermal radiation at the ambient temperature, and environmental conduction and convection. The thermal balance and net cooling power *P*_cool_ of such a radiative cooler (Fig. 2c) can be described as:

1 $$P_{cool}\left( {{{\mathrm{T}}}} \right) = P_{rad} - P_{sun} - P_{atm} - P_{non - rad}$$

$P_{rad}$ can be calculated as:

2 $$P_{rad}\left( {{{{\mathrm{T}}}}_s} \right) = A{\int} {d{{\Omega }}\,cos\theta } {\int}_0^\infty {I_{bb}(\lambda ,T_s)\varepsilon _s(\lambda ,\theta )d\lambda }$$

where ${\int} {d{{\Omega }}\,cos\theta }$ denotes the integration over a hemisphere; $\varepsilon _s(\lambda ,\theta )$ is the emissivity of wavelength *λ* and angle *θ* dependence; and $I_{bb}\left( {T,\lambda } \right) = \frac{{2hc^2}}{{\lambda ^5}}\frac{1}{{e^{hc/(\lambda k_BT)} - 1}}$ is the blackbody spectral emission at temperature *T* where $h = 6.626 \times 10^{ - 34}Js$ is the Planck’s constant, $k_B = 1.381 \times 10^{ - 23}J/K$ is the Boltzmann constant, $c = 2.988 \times 10^8{{{\mathrm{m}}}}/{{{\mathrm{s}}}}$ is the speed of light.

The heat flux of downward radiation from the atmosphere is described as:

3 $$P_{atm}\left( {T_{amb}} \right) = A{\int} {d{{\Omega }}\,cos\theta } {\int}_0^\infty {I_{bb}} (\lambda ,T_{amb})\varepsilon _{atm}(\lambda ,\theta )\varepsilon _s(\lambda ,\theta )d\lambda$$

where $\varepsilon _{atm}(\lambda ,\theta )$ is the emissivity of the atmosphere at wavelength *λ* and angle *θ*.

The absorbed solar power is calculated as:

4 $$P_{sun}\left( {{{\mathrm{T}}}} \right) = A{\int}_0^\infty {\varepsilon _s\left( {\lambda ,\theta _{sun}} \right)I_{sun}\left( \lambda \right)d\lambda }$$

where $I_{sun}\left( \lambda \right)$ is the direct solar spectral irradiance.

5 $$P_{non - rad}\left( {{{{\mathrm{T}}}},T_{amb}} \right) = {{{\mathrm{h}}}}A(T_{amb} - T)$$

where *h* is the overall heat coefficient accounting for conduction and convection between the ambient and cooler surfaces.

To enhance the cooling power $P_{cool}$, which is the radiative cooling under the sunlight, the energy amount emitted from the radiative cooler needs to exceed the solar absorbance due to the large power energy density difference between the solar irradiation, AM 1.5G spectrum, and thermal radiation from the Earth in the mid-IR wavelength range at the ambient temperature (Fig. 2d)^26^. In Eqs. (1)–(5), emissivity is the most important factor, especially the emissivity of atmosphere $\varepsilon _{atm}(\lambda ,\theta )$, which sets the limits for the radiative cooler. The atmosphere contains many gases, such as carbon dioxide, water vapor, and ozone, and gas-filled microparticles. By reciprocity, these gases strongly absorb the IR terrestrial thermal radiation and emit the downward radiation to the terrestrial region, preventing the heat from dissipating into outer space. The downward radiation energy is sensitive to the atmospheric condition, which depends on the rate of production of water vapor, carbon dioxide, ozone, and microparticles less than 2.5 µm (Fig. 2e)^26^. The downward radiation increases with a high humidity ratio, resulting in high $\varepsilon _{atm}(\lambda ,\theta )$ in the mid-IR wavelength; indicating the high performance of radiative cooling frequently in arid regions. However, not all wavelengths are affected by gases. The atmosphere has a transparent window that exists in the IR wavelength range of 8–13 µm; here, gases are not absorbed, and downward radiation is not important in this wavelength range. Thus, the radiative cooler should have a unit emissivity $\varepsilon _s(\lambda ,\theta )$ in the 8–13 µm atmospheric window to achieve sub-ambient cooling that reduces $P_{atm}\left( {T_{amb}} \right)$. In addition, the design of radiative cooler accounts for the angular dependence of the emissivity of atmosphere $\varepsilon _{atm}(\lambda ,\theta )$ for minimizing $P_{atm}$^27^. The emissivity of atmosphere is the maximum when *θ* is at 90° in 4–20 µm range and minimum when *θ* is at 0° in the 8–13 µm of the atmospheric window^28,29^. Thus, the radiative cooler can be optimized by tuning the emissivity in consideration of angular dependency of atmospheric radiation $P_{atm}$. In the solar radiation spectrum, the cooler requires a minimum emissivity $\varepsilon _s(\lambda ,\theta )$ with a high reflectivity to minimize the incoming power from the sunlight $P_{sun}\left( {{{\mathrm{T}}}} \right)$.

Regarding the applications where there is a large cooling power at a temperature near the ambient or largely reduced sub-ambient temperature, the surface of the radiative cooler can be designed as two types: a broadband emitter with unity emissivity at all IR wavelengths and a selective emitter with unity emissivity only within the wavelength range of the atmospheric window (Fig. 2f (left))^30^. The surface with the broadband emitter should have high emissivity at all IR wavelengths to achieve a large cooling power. However, according to Kirchhoff’s law, the broadband emitter not only emits the thermal radiation within the atmospheric window but also absorbs downward radiation outside this window; this reciprocity of the radiation effect makes it difficult to achieve a temperature lower than the ambient temperature. Conversely, the selective emitter, which is near unity emissivity only within the atmospheric window, contributes to the low absorption of downward atmospheric radiation. The broadband emitter has a larger net cooling power when the surface temperature is near or above the ambient temperature, while the selective emitter can achieve a much lower surface temperature. A spectral selective emitter (Fig. 2f (left) “the red line”) has an emissivity of unity in the 8–13 μm wavelength range while a broad emitter (Fig. 2f (left) “the green line”) has an emissivity of unity in all wavelength range. The selective emitter shows much lower temperature than the broad emitter (Fig. 2f (right)). Figure 2f was calculated under the condition that the ambient temperature was set to be 27.15 °C in ideal state that solar absorption ($P_{sun} = 0$.), and parasitic heat ($P_{non - rad} = 0$.) were set to be zero, and a transmission spectrum corresponds to the atmosphere with 60% relative humidity.

## Photonic radiative cooling

**Fig. 3:**
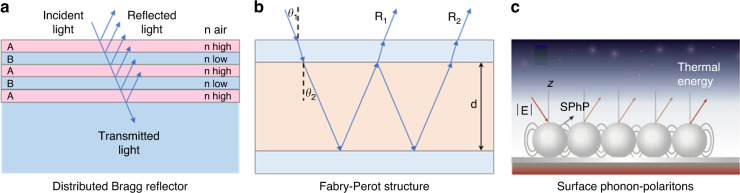
Photonic concepts for radiative cooling. **a** Distributed Bragg Reflector structure **b** Fabry–Perot structure **c** Heat dissipation as thermal energy due to the enhanced emissivity resulting from surface phonon polaritons. Reproduced with permission^33^. Copyright 2019, Wiley-VCH

The ability of photonic structures to manipulate light over a broad range is important for demonstrating radiative cooling concepts. Modifying the thermal emissivity of photonic structures has seen substantial advancement in the pasts. Additionally, the strength of the interaction between light and a structure generally depends significantly on the length scales of the structure. For example, Distributed Bragg reflectors (DBR), in which the reflectivity of the structure is improved by optimizing its optical length, is an important reflector used in multilayered photonic radiative cooling (Fig. 3a)^31^. All the reflected waves undergo constructive interference, producing an overall high reflection, which comprises periodic layers of high and low refractive indices (RIs). The optical length of the structure can be changed by altering the height, RIs, and porosity of the layers. The incident radiation gets reflected well by each layer. The Fabry–Perot cavity of the photonic band structure forms three layers comprising two semitransparent metal layers separated by a dielectric layer (Fig. 3b), which can produce a reflection valley at a certain wavelength and almost perfect absorption^32^. By modifying the thickness and composition of the middle dielectric layer, the resonance wavelength can be changed. This tenability compensates for the weakness in radiative cooling, where energy efficiency decreases due to unilateral heat dissipation in winter. Another process used in photonic radiative cooling is a surface phonon polariton (SPhP), which is a group mechanical oscillation of the polar sphere’s dipoles that interact resonantly with the photon of the same wavelength. A heat source generated by the photon of solar radiation activates SPhP resonances in the polar spheres (Fig. 3c)^33^. By using these characteristics, photonic technology can implement the structures that support high reflectivity and emissivity in the desirable spectrum range. Interestingly, photonic structure helps organisms to regulate their body temperature. Moreover, photonic technology improves the esthetic of color and transparency for broad applications and adds the functionality of dynamic switching to heat and cool based on ambient temperature. In this section, multilayer structure, periodic structure, random structure, bioinspired structure, colored, transparent, and tunable photonic radiative cooler are reviewed in depth.

### Structure of photonic radiative cooling

#### Multilayer structure

**Fig. 4:**
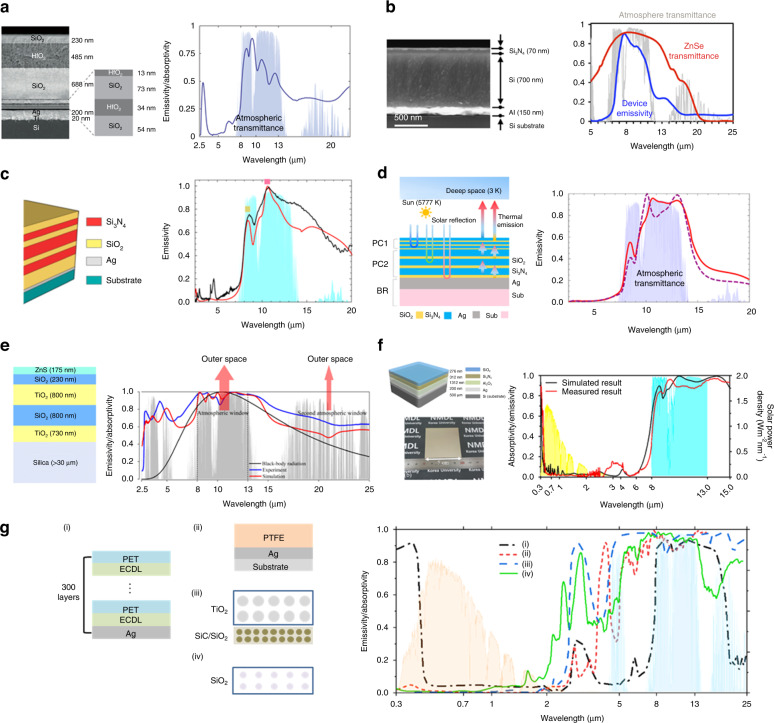
Multilayer structure of daytime radiative coolers. **a** Scanning electron microscope (SEM) image of seven multi layers of SiO_2_ and HfO_2_ on the top of Ag (left) emissivity in the mid-IR wavelengths (right). Reproduced with permission^34^. Copyright 2018, Springer Nature. **b** SEM image of alternating layers of Si and Si_3_N_4_ on the top of Al (left) emissivity of ZnSe in mid-IR wavelengths (red) emissivity of multilayer device in the mid-IR wavelengths (blue) (right). Reproduced with permission^36^. Copyright 2018, Springer Nature. **c** Schematic of multilayer structure (left) measured (black) and calculated (red) emissivity of structure in the mid-IR range (right). Reproduced with permission^37^. Copyright 2020, Elsevier B.V. **d** Schematic of tandem structure (left) emissivity spectra with SiO_2_ (line) and without SiO_2_ (dot) in the mid-IR range (right). Reproduced with permission^38^. Copyright 2019, American Chemical Society. **e** Schematic of multilayer structure (left) simulated (red) and measured (blue) emissivity in the mid-IR range and black body radiation at 300 K (black) (right). Reproduced with permission^47^. Copyright 2018, Optical Society of America. **f** Schematic of multilayer structure and fabricated sample (left) measured (red) and simulated (black) emissivity in the mid-IR range (right). Reproduced with permission^40^. Copyright 2020, American Chemical Society. **g** The spectral emissivity/absorptivity of (i)^48^, (ii)^49^, (iii)^50^, and (iv)^51^. Reproduced with permission^43^. Copyright 2019, AIP

Daytime radiative cooling has been achieved using a recently introduced developing nanophotonic structure^34^. This photonic radiative cooler comprises seven alternating layers of SiO_2_ and hafnium dioxide (HfO_2_) on top of Ag, allowing the reflection of the solar radiation to a level that is achievable utilizing periodic 1-D photonic structure (Fig. 4a (left)). While SiO_2_ is the low-index layer and is optically clear, HfO_2_ acts as a high-index material that also exhibits low ultraviolet absorption, a helpful property when optimizing for solar reflectance. Thermal radiation from the cooler is mainly produced by the top three layers, which are substantially thicker. The SPhP resonance of SiO_2_ creates a strong peak in its absorptivity around 9 µm, and HfO_2_ presents selective emission in the 8–13 µm wavelength range^35^. All these layers work together to exhibits high solar reflectance and strong heat emission, which experimentally showed a temperature reduction of ~5 °C below the ambient under sunlight with 850 W/m^2^ (Fig. 4a (right)). Chen et al.^36^ showed a nanophotonic structure with selective emissivity achieving a temperature reduction of a 60 °C from ambient temperature (Fig. 4b (left)). This structure comprises silicon nitride (Si_3_N_4_), amorphous silicon (Si), and aluminum (Al) on top of an Si wafer. The Si_3_N_4_ layer contributes primarily to the selective emission from the SPhP excitation; Si has an extinction coefficient in the mid-IR wavelength range and the radiative heat loss is suppressed using Al. The emissivity of the structure results in highly selective emission to the atmospheric window (Fig. 4b right). Ma et al.^37^ introduced a photonic structure comprising seven alternating multilayers of SiO_2_ and Si_3_N_4_ to enhance the thermal emission to the atmospheric window. (Fig. 4c (left)). The combination of Si_3_N_4_ with high RI, and SiO_2_ with a low RI facilitates high solar reflection and selective thermal emission within the atmospheric window (Fig. 4c (right)). This radiative cooler achieves a temperature drop of 8 °C below the ambient temperature with 87 W/m^2^ cooling power. The improvement in selective emission using the same combination of materials, namely SiO_2_ and Si_3_N_4_, is demonstrated by the tandem photonic structure (Fig. 4d (left))^38^. The tandem layer stacked with two photonic layers causes an expanded bandgap to approaches the thermal emission peak in the atmospheric window (Fig. 4d (right)) due to the complementary SPhP resonances and high reflection of the ultraviolet radiation, which affects cooling powers. This tandem radiative cooler can yield 11 °C below the ambient temperature under sunlight.

Unlike the selective emitter, some suggested photonic radiative coolers can achieve ultra-broadband absorption with great efficiency due to strongly matching optical resonances. To improve absorption even further, this photonic cooler is composed of the following alternating layers of TiO_2_ and SiO_2_, while the outer layer of zinc sulfide (ZnS) acts as an excellent anti-reflection layer (Fig. 4e (left))^39^. Within the atmospheric transparency window, the combination of all layers increases the average emissivity to 96% (Fig. 4e (right)). The suggested inorganic radiative cooler uses particle swarm optimization and a 1-D matrix formulation for multilayer structures. It has four inorganic layers and one metallic layer (Fig. 4f (left))^40^. In the atmospheric transparency window, the first three layers SiO_2_, Si_3_N_4_, and Al_2_O_3_ produce substantial infrared emission, with peak extinction coefficient values of 9, 11, and 15 µm, respectively. The incoming solar energy is reflected using the layers of metallic Ag mirrors. It is possible to attain high emission values in the atmospheric transparency window while keeping very low absorption in the solar spectrum area using just three layers made entirely of inorganic materials. The cooler combined layers had an average emissivity of 87% in the 8–13 µm range and an average absorptivity of 5.2% (Fig. 4f (right)) in the solar spectral area in the 0.3–2.5 µm range. The cooler has a maximum temperature reduction capability of 8.2 °C.

Polymers and paints appear to have a significant advantage when it comes to production scalability^41–47^. A commercially available polyester reflector with a silver layer decreased the temperature by 2 °C below the ambient under the sun (Fig. 4g (i))^48^. A polytetrafluoroethylene (PTFE) comprising double layers with a silver film at atop showed a high solar reflectance of over 95% and emissivity of over 90% in the mid-IR wavelength (Fig. 4g (ii))^49^. Unlike the polymer film type, particle in polymer was used in radiative cooling. Two layers comprising a top solar reflection layer of TiO_2_ nanoparticle and a bottom layer of SiO_2_ proved a high emissivity in the atmospheric window (Fig. 4g (iii))^50^. A silica microsphere based photonic random medium was introduced for paint application in radiative cooling (Fig. 4g (iv))^51^. The performance of four radiative coolers is presented in Fig. 4g (right).

#### Periodic structure

The role of photonic structures in radiative cooling for controlling thermal emission to the atmospheric window by adjusting the dimensions of periodical nanostructure has also been explored in 2-D and 3-D. Different radiation characteristics are displayed by nanophotonic structures that are close in wavelength^21,52^. Micro-resonator structure are the best in reflecting or absorbing a certain wavelength^53–55^. Daytime radiative coolers have been designed for exploring different nanophotonic structures, including periodic arrays, gratings, conical pillar arrays, metal–dielectric–metal resonators, multilayer pyramidal nanostructures, and dielectric resonator. These structures can be modified to alter the spectrum selectivity.

**Fig. 5:**
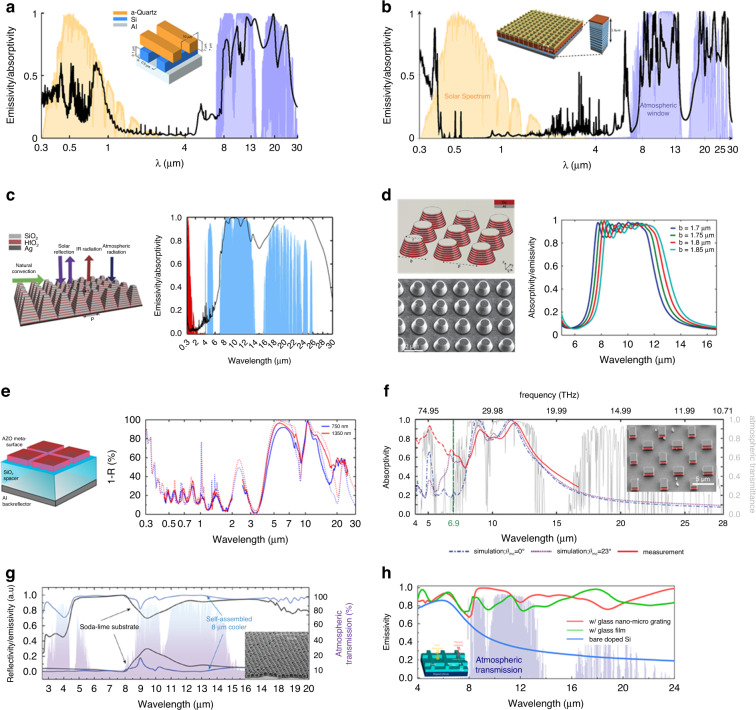
Periodic structure of daytime radiative coolers. **a** Schematic of the radiative cooler structure with quartz bar on top of silicon nanowire (inset) the emissivity/absorptivity spectrum of the combined structure. Reproduced with permission^56^. Copyright 2013, AIP. **b** Schematic of radiative cooler structure comprising SiC and quartz (inset) the emissivity spectrum of the structure. Reproduced with permission^58^. Copyright 2013, ACS. **c** Schematic structure of the pyramidal multilayer (left) simulated emissivity of pyramidal multilayer cooler (right). Reproduced with permission^59^. Copyright 2019, OSA. **d** Design of conical multilayer (left upper) SEM images of conical multilayer (left lower) calculated emissivity of conical multilayer for different bottom diameter (right). Reproduced with permission^65^. Copyright 2015, Wiley-VCH. **e** Schematic of radiative cooler structure (left) Emissivity of the experimental (solid) and simulated (dashed) structure with different lengths, *L* = 750 nm (blue) and 1350 nm (red) (right). Reproduced with permission^66^. Copyright 2017, ACS. **f** SEM image of the fabricated structure (inset), simulated (dot), and measured (line) emissivity of the fabricated structure. Reproduced with permission^67^. Copyright 2017, Wiley-VCH. **g** SEM image of a colloidal crystal of 8 µm sphere (inset) reflectivity and emissivity spectra of soda lime glass (black) and crystal sphere (gray). Reproduced with permission^33^. Copyright 2019, Wiley. **h** Schematic of the nano micro grating structure (inset) thermal emissivity spectra of cooler with glass nano-micro grating (red), with glass film (green), and bare doped Si (black). Reproduced with permission^68^. Copyright 2021, Springer Nature

A periodic array of nanophotnic for color-preserving daytime radiative cooling has been proposed. The structure orthogonally comprises the quartz bar on top of the silicone nanowire and Al at the bottom (Inset Fig. 5a)^56^. Al reflects solar irradiation, and the silicon nanowire produces specific color through it geometric properties^57^. α-quartz was selected for its transparency at solar wavelength, while strongly emitting thermal radiation in the atmospheric window due to SPhP resonance. This structure achieves less solar reflection and more emissivity in thermal radiation to the 1st (8–13 µm) and 2nd (16–25 µm) atmospheric window, where the 2nd atmospheric window also has a radiative cooling power of 10–20 W/m^2^ at the ambient temperature^53^ (Fig. 5a). Rephaeli et al.^58^ showed that a periodic grating structure enhances radiative cooling performance (inset Fig. 5b). The top grating layer as emitter made of α-quartz and SiC due to the phonon-polariton combine photonic reflector of 15 multilayers with TiO_2_ and MgF_2_. The bottom layer comprises periodical layers of TiO_2_ with a high RI, and MgF_2_ with low RI, lying on a silver substrate. The emissivity of the structure is strongly suppressed outside the transparency window and has emissivity peaks in the 8–13, and 20–30 µm range, which covers a secondary atmospheric window (Fig. 5b). This grating nanophotonic radiative cooling showed 100 W/m^2^. A dielectric pyramidal multilayer is presented (Fig. 5c (left))^59^ to achieve daytime cooling performance with high solar reflection and strong IR emissivity within the two atmospheric transparency windows. Two dielectric materials, HfO_2_ and SiO_2_, are chosen for the metamaterial-structure design due to their high transparency in the solar spectrum and high absorptivity loss in the mid-IR region. This photonic structure theoretically provides high cooling performance, leading to a temperature drop of 42 °C with a cooling power of 156 W/m^2^ at the ambient temperature, achieving near-unity selective thermal emission due to the moth eye effect^60–64^. The cooler exhibits extremely low solar absorptivity and high thermal emission (Fig. 5c (right)). A shaped conical pillar structure is achieved by unity thermal emission, which can possess a cooling power of 116 W/m^2^ at the ambient temperature and show the ability to reduce the temperature by 12.2 °C (Fig. 5d (left))^65^. The conical pillar comprising 14 alternating metal–dielectric materials of aluminum and germanium leads to a near-unity emission in the 8–13 µm wavelength range and the emission peak in mid-IR range shifts by a bottom diameters of pillars (Fig. 5d right). A square periodic photonic structure is a possible solution to enhance the radiative cooling performance. The proposed structure with the Salisbury screen comprises a square surface at the top, spacer in the middle, and a reflector on the bottom. (Fig. 5e (left))^66^. The square surface is aluminum doped zinc oxide (ZnO) and the spacer is SiO_2_ enhancing the constructive interference from the reflected radiation at the top layer. This designed structure supports high thermal emission in the atmospheric window (Fig. 5e (right)). A similar square structure can be recommended for the photonic radiative cooling structure. It comprises phosphorous-doped n-type silicon and silver (inset Fig. 5f)^67^. The doped-silicon supports high emissivity in the IR range, while the silver layer enhances the ability to reflect solar irradiation. The data on the emissivity (red line, Fig. 5f) agrees well with those on the atmospheric window (gray line, Fig. 5f). The 2-D spherical periodic grating can contribute to increase broadband emissivity in the mid-IR region. The inset in Fig. 5g^33^ shows a 2-D nanophotonic comprising 8 µm diameter SiO_2_ spheres; these beads enhance the broadband thermal radiation through the SPhP. This spherical grating structure exhibits an averaged thermal emissivity of 98% while the reference substrate without the sphere exhibits one of 88% in the mid-IR region (Fig. 5g) and experimentally achieved a temperature reduction of 14 °C below the ambient. Perrakis et al.^68^ showed that the designed in-plane nano and micro photonic periodic patterns can be utilized in concentrated photovoltaics (CPV) system (inset Fig. 5h). The micro patterned grating enhances the emissivity in the 8–13 µm range as cooling while the nano patterned grating enhances the absorption of optical radiation in the 0.28–1.1 µm to increase CPV efficiency. The combined nano and micro structure still exhibits cooling performance since the optical properties in the micro grating are not affected by nano grating. Hence, the combination of the optimized surface nano and microstructures provides a higher emissivity in the mid-IR (Fig. 5h), and the temperature decreases up to 5.8 °C.

#### Random structure

**Fig. 6:**
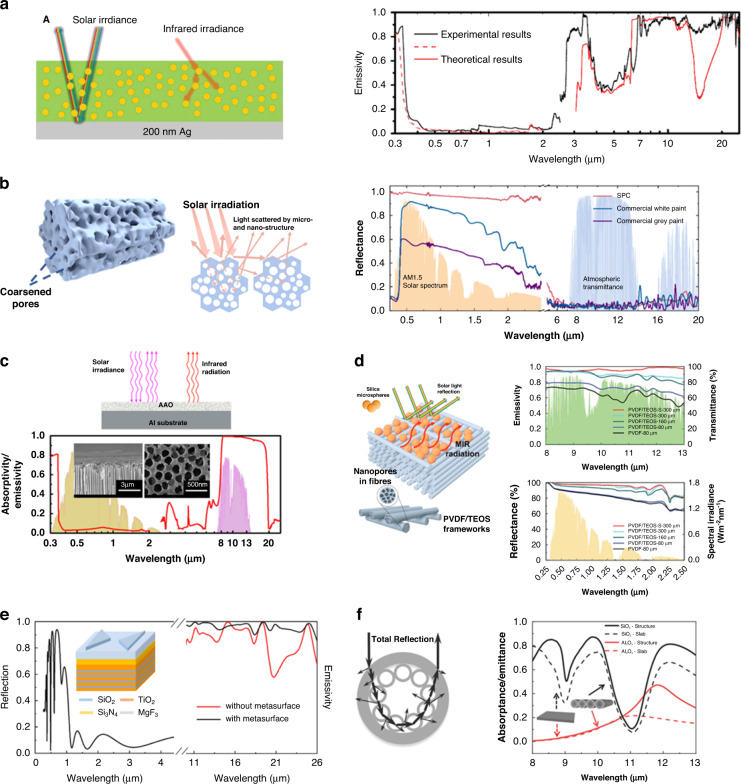
Random structure of daytime radiative coolers. **a** Schematic of the glass particle in the polymer on the Ag (left) thermal emissivity spectra of cooler (right). Reproduced with permission^70^. Copyright 2017, AAAS. **b** Schematic of the fabricated rod-like particle (left) schematic of the light scattering (middle) the reflectivity spectra of particle coating (red) and commercial white (blue) and gray (purple) paint (right). Reproduced with permission^78^. Copyright 2022, Elsevier. **c** Schematic of AAO on the Al substrate (upper) SEM image of section view of AAO and measured emissivity of AAO sample (lower). Reproduced with permission^79^. Copyright 2018, Elsevier B.V **d** Schematic of the nanopore fiber and microsphere (left) emissivity of membrane cooler in mid-IR range (right upper) reflectance of membrane cooler in solar spectrum (right lower). Reproduced with permission^73^. Copyright 2020, Wiley-VCH. **e** Schematic of the ultra-broadband radiative cooler structure (inset) the reflectivity and emissivity spectrum of the cooler with metasurface (black) the emissivity spectrum of the cooler without metasurface (red). Reproduced with permission^89^. Copyright 2021, IOP. **f** Section view of porous cylinder structure (left) simulated emittance spectra of shell and hollow cylinder structure (right). Reproduced with permission^91^. Copyright 2019, The Royal Society of Chemistry

Photonic technology has the unique capacity to modify the spectrum characteristics of the radiative cooling^69^. Thus, it is desirable to use the design of an efficient passive radiative cooling system for daytime usage. A novel configuration of the designed photonic radiative cooler achieves a broadband absorption spectrum, which incorporates a randomly distributed SiO_2_ for the absorption enhanced broadband spectrum^70^. This cooler comprises SiO_2_ micro sphere distributed in a material of polymethyl-pentene (TPX) on Ag (Fig. 6a (left)). The matrix with SiO_2_ sphere does not heat up the material due to low absorption of TPX in visible wavelength and high emissivity in mid-IR due to the strong phonon-polarition resonance of SiO_2_ in the atmospheric window^71^. The combined structure has an average emissivity of 93% in mid-IR range and reflectivity of 96% in visible wavelength (Fig. 6a (right)), exhibiting a cooling power of 93 W/m^2^ in the daytime.

A photonic structure with a rod random pore membrane exhibited a high performance of radiative cooling. Previous research on random pore coating in recent years have already proved a high radiative cooling performance^72–77^. Compared to the coating, paints are more desirable and easier usage for applications, but mostly does not achieve passive radiative cooling due to the poor reflectivity of pigments. A structural in Fig. 6b (left) provides high performance passive cooling paint including rod-like particle^78^. It comprises a micro- and nano-porous structure with a coarsened surface, which contributes to sunlight scattering in the solar radiation range (Fig. 6b (middle)). This structure made of zinc silicate shows purely white due to the scattering of the randomly distributed pores, and enhances highly broad band emission due to abundant the molecular vibrational mode in its material. This cooler demonstrates an excellent solar irradiation reflectance of 97% in the solar range and a thermal emissivity of 95% in the mid-IR range compared to commercial paint (Fig. 6b right), exhibiting an average subambient temperature drop of 4.3 °C in the daytime. A porous cylindrical photonic structure can exhibit a high thermal radiation and low solar absorption (Fig. 6c upper)^79^. The porous alumina (Al_2_O_3_) has a strong phonon resonance in the mid-IR wavelength and a weak solar absorption (Fig. 6c lower), giving rise to a cooling power capacity of 64 W/m^2^, and a temperature drop of 2.6 °C.

The fibers are widely used structure in various fields^80–83^. In radiative cooling field, many researches have shown their usage and performance due to adjustable optical and thermal radiation by diameter and fiber structure^84–88^. A fiber with a sphere-randomly distributed photonic cooler has demonstrated its cooling performance. This fiber hybrid radiator comprises polyvinylidene fluoride/tetraethoxysilane (PVDF/TEOS) fibers with numerous nanopores and SiO_2_ microspheres randomly distributed across their surfaces (Fig. 6d (left))^73^. This photonic structure exhibits an extremely high solar reflectance due to the scattering of nanopores and high thermal emissivity resulting from SPhP resonances of SiO_2_ and the molecular vibrational modes in certain polymer. This cooler has an averaged IR emissivity of 96% and reflects 97% of the solar irradiance (Fig. 6d (right))^73^; and it decreases up to 6 °C under direct sunlight.

A unique grating patterned two triangle of SiO_2_ provides the absorption layer enhanced the broadband spectrum^89^. This cooler comprises a patterned SiO_2_ atop the Si_3_N_4_ and TiO_2_ layers, which behaves as an absorption, and a bottom multilayer alternating nine layers of TiO_2_ and MgF_2_, which acts as a reflector (inset Fig. 6e). Since SiO_2_, Si_3_N_4_, and TiO_2_ has absorption peak ~8–10 µm, 8–14 µm, and 12 µm respectively^90^, the top emitter with pattered surface is designed to increase the emissivity. The optimized patterned top surface enhances the emissivity up to 99% at 9 µm and 98% at 21 µm compared to structure without patterned top surface (Fig. 6e). The average emissivity of the combined structure with patterned surfaces reaches up to 97% in the atmospheric window and the reflection can exceed 93% in the solar band.

Another unique structure shows a radiative cooling effects. This photonic structure comprises a unique shell and hollow cylinder structures comprising an exterior shell and an interior of packed hollow cylinders (Fig. 6f (left))^91^. The shell improved the reflection in the short-wavelength range due to the thin-film interference. The interior optimized the hollow cylinder system, further enhancing the reflection in the long-wavelength range based on Mie resonance and pronounced total reflection. This cooler shows an enhanced thermal radiation emissivity of 7.9% (Fig. 6f (right)).

#### Bioinspired structure

Organisms have evolved to adapt to the unique natural habitat to raises survival possibility to regulate their body temperature for adaptation since temperature is an important environmental factor for them^92^, in which they. As a result, some terrestrial organisms have developed to control their body temperature beneficially^93^. For instance, the presence of hair, and a change in body size to balance the surface-to-volume ratio support the idea that the ability to control body temperature was directly influenced by evolution^94,95^. The Saharan silver ant^96–99^, butterfly^100–105^, beetle^91,106–108^, silk cocoon^109–111^, and others^112–117^ have been reported to exhibit thermal control behaviors. Most natural substances have structural component that help regulate body temperature, since the structures have evolved to deal with reflectivity and radiative heat dissipation.

**Fig. 7:**
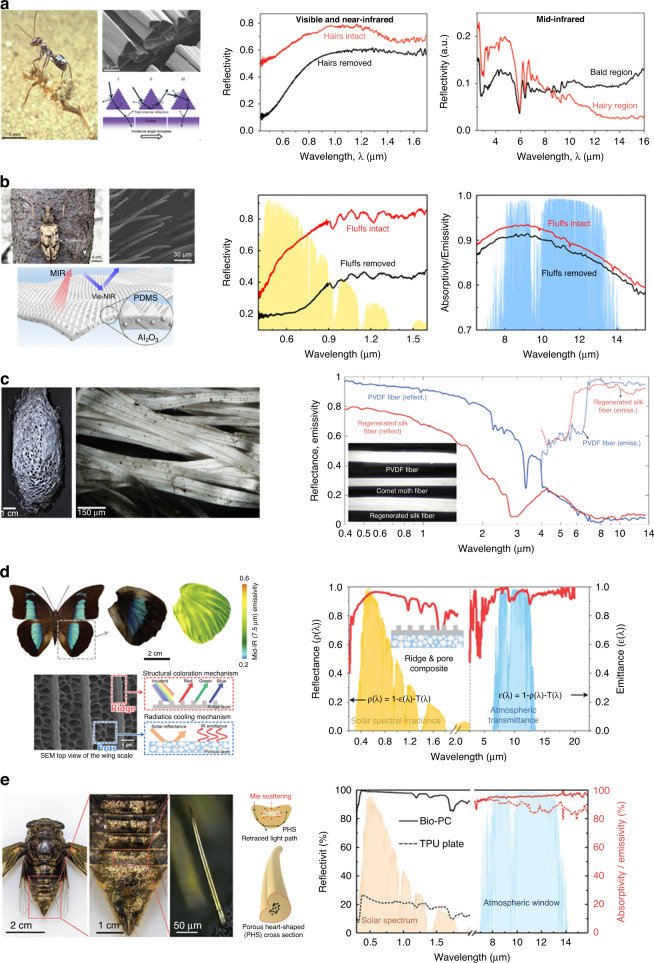
Bioinspired regenerated structure of daytime radiative cooler. **a** Sahara silver ants; SEM and schematic images of the its hairs (left) reflectivity of silver ants body surface with hair (red) and without hair (black) in solar spectrum (middle) the mid-IR range (right). Reproduced with permission^99^. Copyright 2015, AAAS. **b** A male N.gagas and SEM image of its fluffs and bioinspired regenerated radiative cooler (left) reflectivity of silver ants body surface with hair (red) and without hair (black) in solar spectrum (middle) and the mid-IR range (right). Reproduced with permission^106^. Copyright 2021, National Academy of Science. **c** Photograph and microscopic image of cocoon (left) reflectance and emittance spectra of bioinspired regenerated cooler (right). Reproduced with permission^110^. Copyright 2018, Springer Nature. **d** Photograph of a male Archaeoprepona demophon and SEM image of the its grating and pore structure (left); reflectance and emittance spectra of bioinspired regenerated cooler (right). Reproduced with permission^103^. Copyright 2022, RSC. **e** Photograph of a female C.atrata and cross section view of microspike (left) measured reflectivity and emissivity of bioinspired regenerated cooler and TPU film as reference (right). Reproduced with permission^112^. Copyright 2021, Wiley-VCH

The Saharan silver ants can regulate their body temperature under direct sunlight. They are covered with a dense array of triangularly shaped hairs on their bodies, and the triangular hair structure inspired by the ants shows diffuse reflection at incidence angles. (Fig. 7a (left))^99^ This improvement is the result of Mie scattering by the triangular hairs, which reradiated heat in all directions^118–120^. By reflecting the sunlight spectrum and emitting heat, these hairs shield the ants and allow them to maintain lower and steady-state body temperatures. These unique hairs enhance the reflectivity by 64% within 0.4–1.7 µm of the solar spectrum (Fig. 7a (middle)) and emissivity of 15% in the mid-IR in 2.5–16 µm (Fig. 7a (right)); this supports their ability to offload excess heat. They have developed an efficient way to reduce heat absorption from the external environment and simultaneously dissipate excess heat to reduce their body temperature efficiently.

All organisms, not just those living in extreme conditions manage their body temperature. Similar to the Saharan silver ants, the beetles survive in hot areas with temperatures up to 40 °C^121^. The beetles that mostly live in the woods also control their temperature. The corrugated facets of specimens of *N. gigas* with the tapered fluffs are shown in Fig. 7b (left upper)^106^. The dual-scale triangular fluffs enable tropical beetles to decrease their body temperature by increasing the reflectivity in the visible and near-IR (NIR) region, based on total internal reflection and Mie scattering; enhancing mid-IR emissivity to radiate heat to the surroundings. Taking inspiration from the beetle, bioinspired radiative cooling film is regenerated (Fig. 7b (left lower)). This film comprises spherical particles of Al_2_O_3_ embedded in periodic pyramid fluffed PDMS. These dual-scale microstructures effectively increase reflectivity up to 95% in the solar spectral range (Fig. 7b (middle)) and enhanced emissivity over 96% in the mid-IR spectral range (Fig. 7b right), where cooling power of about 91 W/m^2^ and temperature reduction of 5 °C was measured. The combine effects decreased the beetle’s body temperature, allowing it to survive at high environmental temperatures.

The cocoons made by random structure of silk moths fiber and sericin shields pupae from overheating under the sun, and external impact^122–126^. The cocoons and their individual silk threads display a bright, and silvery metallic shine when exposed to sunlight (Fig. 7c left)^110^ due to the increase in reflectance at visible wavelengths results from numerous light-scattering interactions between the random voids inside the fibers, where the void sizes are similar to those of sunlight. The scattering of incoming light, generated by the random voids inside the fibers, increases the reflectance of the fiber and makes them high absorption in the mid-IR region between 6 and 14 µm because of a strong and wide chemical bond absorption of proteins. The inset Fig. 7c shows bioinspired radiative cooling fiber made of PVDF, and this biomimetic fiber has strong back-scattering in the visible and NIR regions, thus enhancing the solar reflectance to 93% and thermal emissivity to 91% in the mid-IR region (Fig. 7c right “red”)^110^. Thus this combined effects help silkworm cocoons maintain their body temperature and turn into moths in direct sunlight.

Most butterflies must keep their body temperature of 20–50 °C to fly^127–129^. Butterflies may have evolved their wings that change their solar absorptivity and mid-IR emissivity, to successfully regulate the temperature of their body^105,130,131^. Hence their wings have nanostructures dissipating heat and preventing body temperature over 50 °C^132–134^. For example, the butterfly, Archaeoprepona demophon, has wings to regulate its body temperature using a nano porous structure^103^. Their wings in a specific region have a distinctive nanostructure, which enhances their thermal emissivity in the 7.5–14 µm range and allows them to dissipate heat, and a high reflectivity for reducing solar absorption and cool their wings (Fig. 7d (left)). The bioinspired radiative cooler of the butterfly wings, based on periodic grating at the top and nanoporous layer made of polyvinylidene fluoride cohexafluoropropylene (PVDF-HFP), exhibit high reflectance in the solar range and high emittance in the IR atmospheric window (Fig. 7d (right)). The biomimetic film exhibited a high solar reflectance of 94% and thermal emissivity of 96%, resulting in a maximum temperature reduction of 8.45 °C. The results highlight the impact of nanostructures on the mid-IR optical characteristics of butterfly wings and their effects on thermoregulation.

The cicada, Cryptotympana atrata, possesses microspikes with a nanophotonic porous heart-shape (Fig. 7e (left))^112^. The microspikes in the hairy area have radiative cooling properties that protect the cicada’s body from overheating in hot summers. Taking inspiration from the microspikes, photonic emitter is fabricated with alumina nanoparticle embedded in porous thermoplastic polyurethane. The regenerated photonic radiative cooler exhibits a high reflectivity of 98% for solar irradiance due to efficient the Mie scattering by the microscale pores; and an average emissivity as high as 95% in the atmospheric window owing to the antireflection capability of the microhumps and phonon polarization resonances in the mid-IR range (Fig. 7e right)^112^. A maximum temperature reduction of 6.6 °C and cooling power of 78 W/m^2^ at around solar noon was experimentally demonstrated. Different from others, some bioinspired photonic radiative cooling structures can enhance radiative cooling in the daytime^101,135–137^.

### Functionality of photonic radiative cooling

#### Colored functionality

The radiative cooling-based design maximize its performance mainly using metal mirrors or white materials with high solar reflectance. Such materials are frequently used in radiative cooling system designs to enhance cooling performance. Their limited use in practical applications is due to their broadband reflectance at visible wavelengths. For esthetic or practical reasons, white hues, for example, are frequently undesirable as coatings on structures or other items^138–140^. Additionally, the white or silvery glare from these patterns can damage human eyes. To solve this problem, colored photonic radiative coolers have been investigated^21,141–150^. This section introduces the colored photonic and opals used in radiative cooling.

**Fig. 8:**
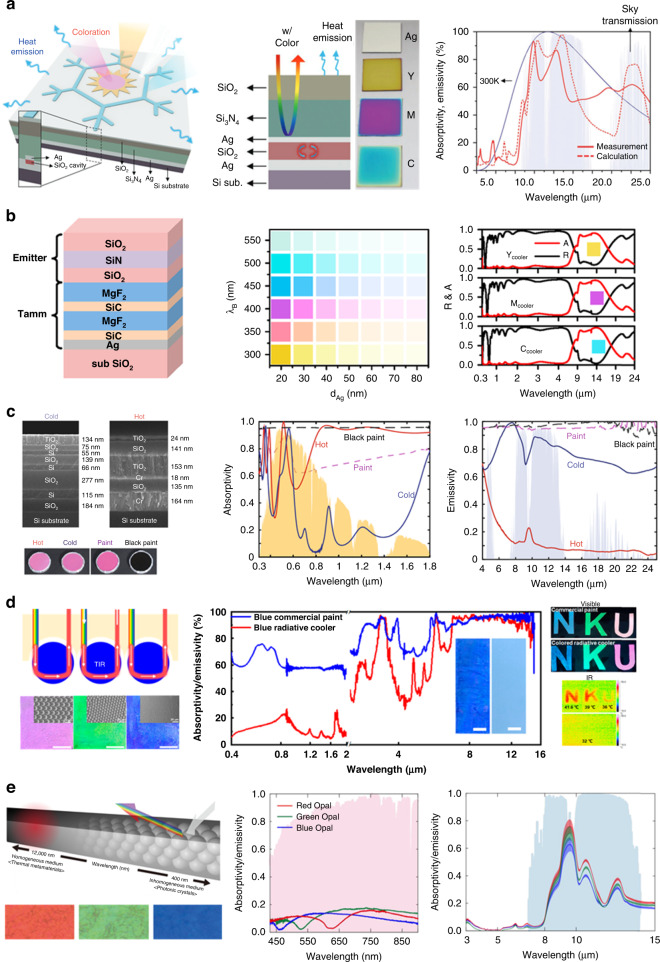
Colored photonic radiative coolers. **a** Schematic of the colored passive radiative cooler (left) section view of colored passive radiative cooler (middle) calculated (dash line) and measured (line) emissivity in mid-IR range (right). Reproduced with permission^141^. Copyright 2018, Wiley-VCH. **b** Schematic structure of the designed colored radiative cooler (left) displayed color by adjusting thickness of silver and DBR (middle) emissivity (red) reflectivity (black) of three colors in the mid-IR range (right). Reproduced with permission^142^. Copyright 2019, ACS publications. **c** SEM image of the “cold” and “hot” structures of same color (left) absorptivity spectra of ‘cold’ (blue line) and “hot” (red line) with the pink (pink dashed) black (black dashed) paints (middle) emissivity spectra of cold’ (blue line) and ‘hot’ (red line) with the pink (pink dashed) and black (black dashed) paints (right). Reproduced with permission^21^. Copyright 2018, Springer Nature. **d** Schematic of retroreflection-induced color with PS microsphere of 15 (red), 8 (green), 3 (blue) um respectively (left) emissivity spectra of blue colored cooler and blue commercial paint (middle) optical and IR image of “KNU” (right). Reproduced with permission^144^. Copyright 2022, ACS publication. **e** Schematic of opal colored radiative cooler with silica nanosphere of 290 (red), 240 (green), 290 (blue) nm (left) emissivity spectra of opals in the solar (middle) and mid-IR (right) ranges. Reproduced with permission^143^. Copyright 2020, American Chemical Society

Figure 8a (left)^141^ shows the colored radiative cooling of a photonic nanostructure for esthetic purposes. This nanostructure comprises mainly three parts; selective emitters to emit the heat; a solar reflector in the solar spectrum regions, and a metal–insulator–metal (MIM) for creating a vivid color combination of cyan, magenta, and yellow. The selective emitter, which is the top layer, comprises a bilayer of SiO_2_ and Si_3_N_4_, and the solar reflector below the emitter comprises an Ag film. The MIM structure with Ag-SiO_2_-Ag is located on the bottom layer; color is generated by tuning the cavity thickness between Ag and SiO_2_ for cyan, magenta, and yellow respectively (Fig. 8a (middle)). The designed selective emitter with SiO_2_ and Si_3_N_4_, which possesses the peak in the atmospheric window to dissipate heat efficiently, consists with measurement (Fig. 8a right)^141^. Thus, the combined photonic cooler of top emitter, colored generation cavity, and Ag reflector demonstrated a temperature reduction of 3.9 °C below the ambient. Sheng et al.^142^ introduced another colored multilayer cooler by Tamm structure, which is fabricated between a DBR and a metallic substrate^151^. This multilayer structure comprises the Tamm structure of two pairs of MgF_2_ and SiC as the DBR on top of a Ag film, and the emitter comprising three layers of SiO_2_ and silicon nitride (SiN) (Fig. 8b (left)). The hue of the colors displaying on the cooler can be controlled by adjusting the thickness of the DBR and Ag. The thickness of DBR determines color hue and the thickness of Ag layer controls the color purity (Fig. 8b middle). The radiative emission and reflection spectra of the combined coolers provides high reflectance in the solar irradiation and thermal emission spectral band of 8–20 μm with displaying the colors (Fig. 8b right)^142^. This Tamm photonic cooler achieved a cooling power of up to 52 W/m^2^ and a temperature reduction of up to 6 °C at the ambient temperature. Li et al.^21^ showed two photonic multilayer structures that exhibit the same pink colors but differ significantly in their radiative thermal loads. For the “cold” photonic structure, it comprises seven alternating layers of Si and SiO_2_ and a layer of TiO_2_ as the top layer (Fig. 8c left “cold”). The contrast in RI between the Si and SiO_2_ provides the high reflection in the NIR region, a useful feature to minimize the absorption in solar spectrum regions (Fig. 8c (middle) “cold”). The combination of SiO_2_ and TiO_2_ with properly designed thicknesses mainly supports the large thermal emissivity in the mid-IR wavelength range to emit the heat (Fig. 8c (right), “cold”). The “hot” photonic structure comprises three layers of MIM with chromium (Cr) as the metal component on the bottom and three dielectric layers on the top (Fig. 8c left upper “hot”). This “hot” structure shows high absorption in the NIR region, while indicating the low emission in the mid-IR range due to the Cr with high solar absorption and; strongly suppressed thermal emissivity (Fig. 8c (middle and right), “hot”). The combination of the material properties and thickness of the layer further allows the fine tuning of the visible reflection spectrum to achieve the desired color, despite the different thermal properties. The “cold” photonic structure shows low-temperature difference of 15 °C compared with the same pink color paint, while the “hot” photonic structure exhibits about 7 °C higher than the black paint. Thus, with photonic engineering, the two photonic structures exhibit spectral properties that differ significantly from those of conventional color paints.

The diverse structural coloration is produced by microscopically structured surfaces in nature, such as opals^152^. Unlike planar photonic multilayer structures, opal structures are generally a close-packed face-centered-cubic of a 3-D periodic structure, where incoming visible light has reflection peak to color by Bragg diffraction^153^. Yu et al.^144^ show that the opal structure of a colored radiative cooler by retroreflective structural color. The cooler comprises a polystyrene (PS) microsphere on the transparent tape as a top layer that produces structural color and a PDMS on Al foil as a bottom layer that enhances IR radiation while reflecting the rest of the solar irradiation. The optical propagation from PS microsphere to air contributes to total internal reflection in microsphere, which causes the Bragg diffractive structural color of retroreflective light^154–156^; the three colors could be modulated by the diameter of the microspheres (Fig. 8d (left)). The sets of spectral comparisons between radiative coolers and commercial paints with different colors were performed (Fig. 8d (middle)). Two visible images of “NKU” in the commercial and photonic cooler can vividly be seen. Their IR image shows that the pattern in the commercial cooler is hotter than that in the photonic cooler (Fig. 8d (right)). This colored cooler experimentally demonstrates that the temperature always decreases by 4 °C under sunlight whereas commercial paint is always hotter than ambient up to 27 °C in blue color. Another opal photonic radiative cooler demonstrated refractive color preservation. This cooler comprises silica nanospheres as top layer and a PDMS layer on the crystalline Si wafer at the bottom^143^. By adjusting the size of nanospheres opals at the top layer exhibits red, green, and blue colors by Bragg diffraction (Fig. 8e (left)). The silica opals cooler experimentally determined the absorptivity and emissivity in the visible and mid-IR areas (Fig. 8e (middle, right)), and decrease the temperature difference of 15 °C between the opal color of red and Si wafer under the solar light, maintaining its non-absorbing colorization.

#### Transparent functionality

The appearance of a radiative cooler is important in improving its practicality. The high reflectance in the visible range makes most radiative coolers opaque and appear white or silver. To enhance the esthetic impression of coolers for applications, some designs for colored radiative cooling technologies were discussed in the previous section. However, despite the significant improvements in the esthetic impressions of radiative cooler for various applications, there are still restrictions on the structure such as windows and the exterior of compounded colors. Transparent radiative cooling can have a potentially high impact on their cooling efficiency since the window is the main factor in increasing the cooling load in the automobile and building field^157^. Generally, the windows of vehicles and buildings absorb 20–95% of incoming sunlight^158^, and their interior temperatures can reach over 60 °C and 35 °C, respectively. Therefore, an advanced radiative cooling design for transparent functions with simultaneous thermal regulation is needed. Some researches of transparent radiative cooling technologies such as nanospheres^159,160^, nanowire^160–162^, and photonic crystal^163,164^ have been demonstrated, and this section introduces multilayer^165–167^ and micro spherical photonic^168,169^ used in transparent radiative cooling.

**Fig. 9:**
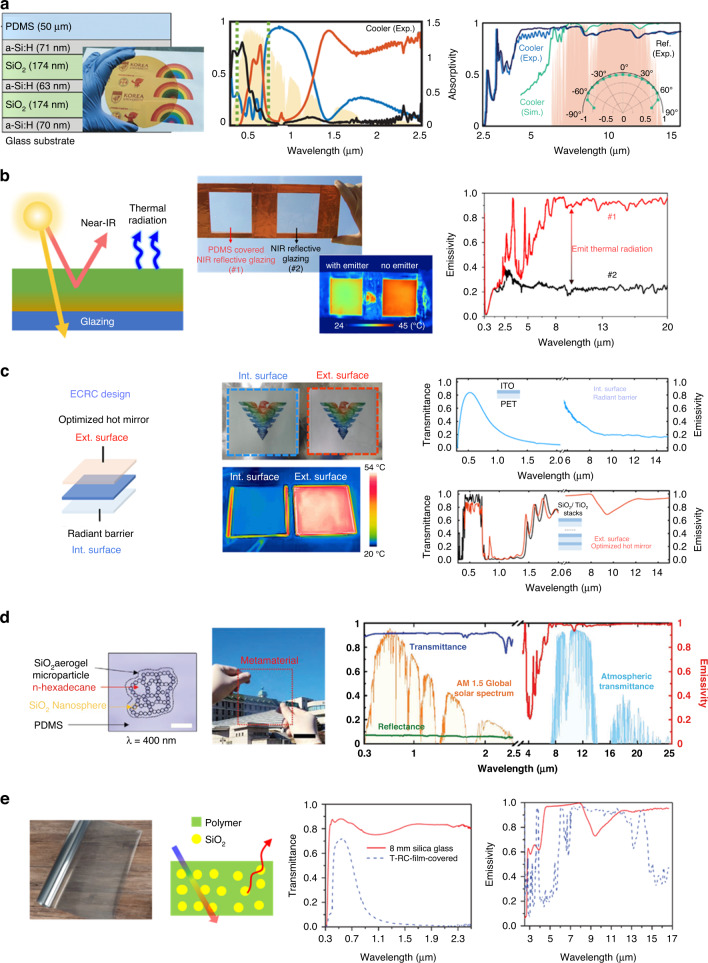
Transparent radiative coolers. **a** Schematic design and optical image of fabricated of a transparent radiative cooler (left) measured emissivity (black), transmittance (red), and reflectance (blue) spectra of the cooler in solar irradiation range (middle) measured emissivity of the cooler in the mid-IR range (right). Reproduced with permission^166^. Copyright 2021, Wiley-VCH. **b** Schematic structure of a transparent radiative cooler (left) optical and IR image of NIR reflection with and without PDMS (middle) measured emissivity spectra of #1 and #2 in mid-IR range (right). Reproduced with permission^165^. Copyright 2020, Elsevier. **c** Schematic deign of enhanced colored-preserving radiative cooling (ECRC) (left) optical and IR image of interior and exterior surfaces (middle) transmissivity and emissivity spectra of exterior surface (right upper) and interior surface (right lower). Reproduced with permission^167^. Copyright 2022, Springer Nature. **d** Simulated 2D model with n-hexadecane infiltration (left) optical image of fabricated transparent cooler (middle) measured transmissivity and reflectivity of transparent cooler (right). Reproduced with permission^168^. Copyright 2022, Wiley-VCH. **e** Optical image and schematic structure of transparent radiative cooler (left) transmissivity and emissivity of 8 mm glass and transparent cooler in the solar spectrum (middle) and mid-IR (right). Reproduced with permission^169^. Copyright 2021, KeAi

For transparency and cooling under the sunlight, the material has to transmit solar irradiation in the visible wavelength range and reflect solar irradiation in the NIR, accounting for 51% of the total solar energy^170,171^, while emitting the heat in the mid-IR wavelength. Hence, the simultaneous control of three regions, visible, NIR, and mid-IR wavelength requires in higher photonic technologies. Kim et al.^166^ introduce a transparent cooler comprising a PDMS top layer and a dielectric of five multilayers of hydrogenated amorphous silicon (a-Si:H) and SiO_2_ stacked alternatively on a glass substrate (Fig. 9a left). The fabricated transparent cooler transmits the back side of the rainbow photo and letter; it is yellow due to the low transmittance in the blue range (inset Fig. 9a (left)). The a-Si:H and SiO_2_ multilayers, which act as DBR, are designed to transmit the visible radiation while reflecting NIR within 0.74–1.4 μm, and the top PDMS layer contributes to selective emissivity in the atmospheric window region as a consequence of optical phonon resonance^172^. The combined cooler is optimized to maximize the emissivity shows the selective emitter in the atmospheric window region, and the transmittance in the visible region while reflecting in the NIR (Fig. 9a (middle and right)). Outdoor rooftop experiments demonstrated that this colored cooler reduced the system interior temperature by an average 5.2 °C. Zhou et al.^165^ introduced a simple double-layered structure of a transparent radiative cooling window transmitting visible light and reflecting the NIR while emitting thermal radiation (Fig. 9b (left)). This transparent cooler uses two materials of indium tin oxide (ITO) and PDMS. ITO, a well-known transparent metal oxide, has optical properties of the reflection of the NIR radiation and low emission in mid-IR range due to the relatively high plasma frequency. Thus, another transparent layer is needed for emitting heat to the atmospheric window in mid-IR range. PDMS is a good candidate for the transparent emitter with the methyl group and the vinyl-terminated cross-linkers bonded on the chains, which reduce reflection in the atmospheric window. Figure 9b (middle upper) shows two different glazing types: a PDMS coating on ITO sample #1, and a no coating on ITO sample #2. The IR camera image shows a much lower surface temperature for sample #1 than it does for sample #2 (Fig. 9b (middle lower)). Both samples produce optically similar results in reflecting high NIR, but sample #1 with PDMS top layer has enhanced higher emissivity in the mid-IR (Fig. 9b (right)). This cooler can decrease the temperature of up to 7 °C and the cooling energy can be saved up to 49 MJ/m^2^ per year. Another photonic multilayer structure for transparent radiative cooling is two photonic films with interior and exterior surfaces, called an enhanced color-preserving temperature cooling (Fig. 9c left)^167^. The transparent exterior surface, acting as the top layer, contributes to a reflection in the NIR, causing the exterior surface to reduce solar irradiation and a broadband emissivity in the mid-IR dissipating heat from the cooler; the transparent interior surface at the bottom reflects the mid-IR minimizing the heating load. In the optical visualization and the IR camera, the film covered by the interior surface shows transparency in the visible range and a low radiation temperature in IR camera, indicating suppressed heat emission; in contrast, the film covered by the exterior surface shows a transparency in the visible range and a high radiation temperature in the IR camera, revealing large radiant energy (Fig. 9c (middle)). The interior surface is a thin layer of transparent ITO on a polyester film having a low mid-IR emissivity in the mid-IR band due to the high carrier concentration of the ITO film, and a high transmissivity in the visible region (Fig. 9c (right upper)). The exterior surface with the periodic nanostructure consisting of 30 layers of TiO_2_ and SiO_2_ provides high visible transmittance, high NIR reflectance, and high broadband mid-IR emissivity (Fig. 9c right lower)^167^. This cooler experimentally demonstrated an enclosed system with power saving up to 63% when the ambient temperature is ~26 °C.

Unlike the multilayer photonic structure of transparent radiative cooling, micro sphere of photonic cooler can be used. In this microsphere, SiO_2_ aerogel micro particles comprising n-hexadecane surrounded with SiO_2_ nanoparticles are randomly distributed in PDMS (Fig. 9d (left))^168^. The micro size of SiO_2_ particles randomly infiltrated in the polymer becomes visibly translucent. However, SiO_2_ micro particles of n-hexadecane in PDMS appear transparent due to their similar RI in the visible wavelength range^46,173,174^ (Fig. 9d (middle)). SiO_2_ also affects the enhancement of the mid-IR by the phonon-polariton resonance of Si-O bonds^51,70,73^, and induced Mie scattering^73,75^. N-hexadecane containing C-C and C-H bonds increases the emittance of light at 3–4 and 7–15 μm^75^. Therefore, this metamaterial cooler with 30 μm thickness provided high transmittance of over 91% in the visible wavelength and high emissivity of over 98% in the atmospheric window (Fig. 9d (right)), and exhibited a solar cell surface temperature reduction of 9.1 °C. Yi et al.^169^ demonstrated energy saving with transparent glass-polymer hybrid radiative cooling made of polyethylene terephthalate wherein silica microspheres were randomly distributed (Fig. 9e (left)). This transparent film is combined with 8 mm thick silica glass since glass pane assists to emit the mid-IR to the outer space. The glass with transparent radiative cooling film shows transmissivity of ~60% in the visible range and an emissivity of over 90% in the mid-IR within the 8–13 μm wavelength range (Fig. 9e (right)). To evaluate the cooling energy-saving efficiency, two identical model boxes with dimensions of 1.0 m × 0.6 m × 1.2 m were built, and the inside air temperatures were recorded. The experiment’s results reveal a maximum temperature difference of 21.6 °C between the two model boxes, demonstrating a reduction of up to 63% in the exhibition building’s annual air conditioning energy consumption.

#### Tunable functionality

Despite the success of radiative cooling technologies in creating esthetic impressions, they could dissipate heat into the universe, even at a temperature of 3 K, producing subambient temperatures of surfaces even in undesirable conditions, such as those during winter. Besides, since the temperature varies seasonally, radiative cooling adopted in the system may increase energy consumption when heating is demanded. Therefore, a radiative cooling system that reduces energy consumption in an environment with any temperature is highly desired. The systems need to maximize their emissivity in the atmosphere window for cooling when the temperature is above a critical value, but minimize emissivity in the entire thermal wavelength for heating when the temperature is below a critical value. Currently, some self-adaptive radiative cooling systems have been reported, which exist in various types^175^: modulating cooling and heating by temperature^176–192^, humidy^74,136,193^, mechanical^194–200^, and electrical^201^ responds. This section introduces temperature based phase change material of vanadium dioxide (VO_2_)^187–192^ and electrical^201^ respond.

**Fig. 10:**
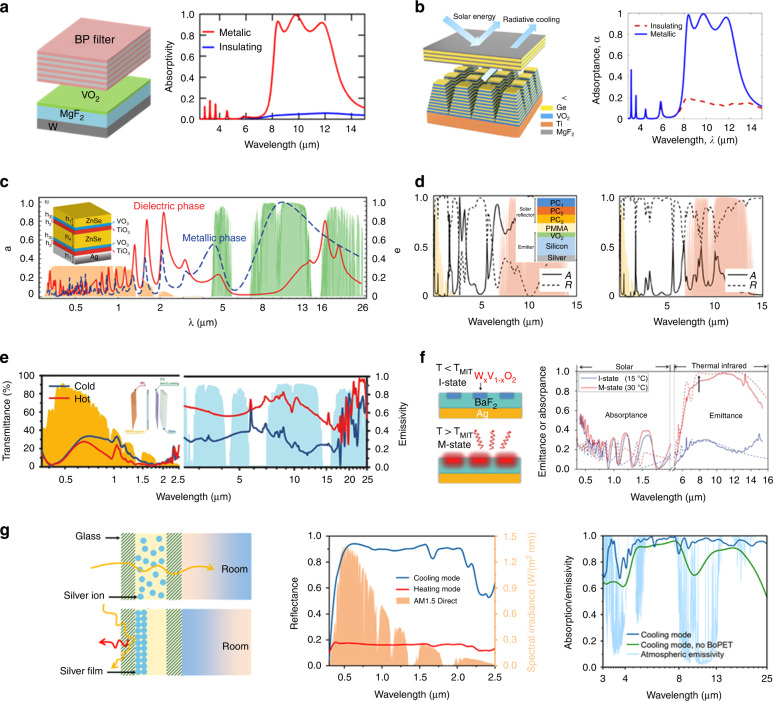
Schematic structures of switchable radiative cooling. **a** Schematic of combined switchable structure comprising bandpass filter on top and radiative cooler on the bottom (left) emissivity spectra of metallic (red) and insulating (blue) in the mid-IR range (right). Reproduced with permission^188^. Copyright 2018, Optica publishing group. **b** Schematic of combined switchable structure comprising filter on top and trapezoidal multilayer cooler on the bottom (left) emissivity spectra of metallic (blue) and insulating (red) in the mid-IR range (right). Reproduced with permission^189^. Copyright 2020, Optica publishing group. **c** Schematic of multinalyer photonic structure (inset) calculated emissivity spectra of dielectric (red) and metallic (blue). Reproduced with permission^187^. Copyright 2018, ACS. **d** Schematic structure of switchable radiative cooler (inset left) emissivity and reflectivity spectra when VO_2_ is in metallic (left) and insulating (right). Reproduced with permission^190^. Copyright 2021, OE. **e** Schematic structure with switchable cooler (inset) regulation of transmissivity and emissivity spectra at 20 °C (cold) and 90 °C (red) Reproduced with permission^192^. Copyright 2021, AAAS. **f** Schematic structure of TARC (left) measured emissivity spectra of TARC at 15 °C (blue line) and 30 °C (red line) with calculated emissivity (dashed line) (right) Reproduced with permission^191^. Copyright 2021, AAAS. **g** Schematic mechanism of switchable radiative cooler in cold (left upper) and hot (left lower) solar reflectivity spectra of glazing window in heating (red) and cooling (blue) (middle) emissivity spectra of glazing window with (blue) and without (green) BoPET film in cooling (right) Reproduced with permission^201^. Copyright 2022, Elsevier

A photonic structure comprises two components, namely a selective emitting band pass filter (BP filter) in the mid-IR range on the top, and a switchable radiative cooling system that provides temperature adaptive radiative cooling at the bottom (Fig. 10a (left))^188^. The BP filter comprises 11 multi-stacked layers of germanium (Ge) and magnesium fluoride (MgF_2_) and is designed to block solar irradiation, while allowing only wavelengths in the 8–13 μm range of the atmospheric window to be transmitted. The temperature adaptive layer comprises VO_2_, MgF_2_, and tungsten (W), which shows Fabry–Perot resonance and forms a cavity in the dielectric layer at least a quarter wave in thickness between the two high reflectors^202–205^. When the ambient temperature rises or falls above the phase transition temperature, VO_2_ exhibits a metallic or insulating state. It exhibits high emissivity, acting as a Salisbury screen absorber^206^ in the metal state, and MgF_2_ acts as cavity, producing the resonant wavelength to absorb the rest of the wavelength from the VO_2_ layer. On the other hand, VO_2_ strongly prevents emissivity in mid-IR range. Thus, when the two components are combined, in the metallic state of the temperature adaptive layer, the system barely absorbs the solar radiation in the solar wavelength range, and provides a strong selective emissivity from 8–13 μm in the IR wavelength range (Fig. 10a “metallic”). Radiative cooling is off when the system is below the critical temperature and VO_2_ is in the insulating state, as shown by the observation of minimal emissivity. (Fig. 10a “insulating”). Zhang et al.^189^ also presented a temperature dependent radiative cooling system. This cooling system comprises a top square filter and a periodic trapezoidal structure at the bottom (Fig. 10b (left)). The filter comprising 11 layers of Ge and MgF_2_ enables the cooler to reflect most of the solar irradiation during daytime and transmit only wavelength of 8–13 μm. The 3D periodic trapezoidal structure comprising 50 layers of VO_2_ and Ge on titanium (Ti) substrate exhibits low absorption of solar irradiation in the wavelength between 0.5 and 2.5 μm and tunable thermal emissivity in the wavelength of 8–13 μm based on VO_2_ temperature. When the temperature reaches to point of phase transition to metallic status, the average emissivity in the wavelength of 8–13 μm increases up to 85%, whereas when the temperature is below the point of phase transition to insulating status the average emissivity in the mid-IR range mostly decreases down to 20% (Fig. 10b (right)). Kort-Kamp^187^ et al. proposed a VO_2_-based tandem structure of radiative cooling tunable in the mid-IR wavelength range. This cooler comprises alternating six layers of TiO_2_, VO_2_, and ZnSe that provide selective emissivity through the atmospheric window and a Ag layer that prevents solar spectrum wavelength range at the bottom (inset Fig. 10c). To be tunable in the mid-IR range, one transparent layer has to be filled in between two mirror layers, which forms a Fabry-Perot cavity for resonation at mid-IR wavelengths. ZnSe is suitable to fill the cavity due to its high transparency in the visible to the mid-IR wavelength range. In mid-IR range, when VO_2_ is in the dielectric phase, the emissivity decreases since the VO_2_ and filling layers are transparent in the wavelength range, resulting in high reflectance from the back scattering of the Ag layer. Conversely, when VO_2_ is in the metallic phase, the emissivity increases due to the resonance of the filling and metallic VO_2_ layer (Fig. 10c). The passive tunable cooler provides temperature reduction of 6 °C below the ambient temperature in summer and an increase of 11 °C above ambient temperature in winter. Similar multilayer, Kim et al.^190^ introduced tunable radiative cooler inserting a spacer between a solar reflector and a emitter. The reflector at a top layer, reflecting solar radiation, comprise three photonic crystals as DBR^207^ in the 0.5–1.2 μm range, and the emitter as a top layer comprises three layers of silver, silicon, and VO_2_ (inset Fig. 10d (left)). When the emitter temperature is above the critical temperature, it turns into metallic state resulting from The Fabry–Perot resonance due to the cavity, and high emissivity in the mid-IR range is presented (Fig. 10d (left)). In contrast, when the emitter temperature is below the critical temperature, the emitter turns as an insulator resulting in less emissivity in the mid-IR range (Fig. 10d (right)). The radiative cooling regulating thermochromics (RCRT)^192^ system comprises three layers of VO_2_, PMMA, and ITO on glass (inset Fig. 10e). In this system, the three layers in each layer have a role to perform Fabry–Perot resonance^208^; VO_2_ induces the temperature phase change of the insulator for undergoing metallic transformation; PMMA supports high transitivity in the solar and mid-IR wavelength range; ITO shows high transitivity in the visible wavelength range but low emissivity in the IR wavelength range. Depending on the transition state of VO_2_ at the critical temperature of 90 °C, the RCRT has a low emissivity at the insulation state of VO_2_ below the critical temperature while strongly radiating heat above the critical temperature at the metallic state of VO_2_ in the mid-IR region^209^. The RCRT system provides a higher emissivity in the “hot” state than in the “cold” state in the mid-IR region, and a higher transmissivity in the “cold” state than the “hot” state in the solar spectrum, allowing more heat inside for warming (Fig. 10e). This passive tunable cooler yielded energy saving up to 325 MJ/m^2^. Tang et al.^191^ introduced a flexible coating structure for temperature-adaptive radiative coating (TARC). This coating has thin patterned W_1.5_V_0.5_O_2_ blocks embedded in the BaF_2_ layer on top of the Ag film (Fig. 10f (left)). Similar to the phase change property of VO_2_, this doping W_1.5_V_0.5_O_2_ exists as a metal or insulator but the metal–insulator transition temperature is reduced to ~22 °C^210^. These three composition layers can induce Fabry–Perot resonance when the thickness of BaF_2_ has a quarter-wavelength cavity structure^188,209^. When the environmental temperature is lower than the transition temperature of the material, which indicates the insulating (I) state, the material barely absorbs the mid-IR wavelength since the wavelength transmitted via W_1.5_V_0.5_O_2_ is reflected by the Ag mirror with little absorption^34^ (Fig. 10f (right) “I-state”). On the other hand, the material mostly emits mid-IR wavelengths when the ambient temperature exceeds the material’s transition temperature, which denotes the metallic (M) state^34^ (Fig. 10f (right) “M-state”). TARC demonstrates thermal emissivity and temperature switches up to 90% and 20 °C, respectively.

Although the thermochromics material of VO_2_ has the benefit of no external energy usage to switch between the cooling and heating states, it cannot reflect consumer demand when required. Unlike the temperature respond switchable cooler using VO_2_, Zhao et al.^201^ presented dynamic glazing panel that can switch between solar heating and radiative cooling modes based on the user’s demand. Two transparent, ITO-coated glasses with ~1 mm thickness make up the dynamic glazing panel, which is sandwiched between the layers of transparent electrolyte that contains silver ions (Fig. 10g (left)). In less than 300 s, the glazing panel may change from a heating mode to a cooling one. When a low voltage of ~2.5 V is used to tint the panel, the glazing panel shifts to radiative cooling mode, which reflects 89% of the incoming solar energy (Fig. 10g (middle), “cooling mode”). In the heating mode, the glazing panel permits ~70% of the incident solar energy to enter the building’s interior (Fig. 10g (middle), “heating mode”). The dynamic glazing panel’s mid infrared emissivity in cooling mode is displayed in (Fig. 10g (right)). The results of the outdoor experiment show that when the ambient temperature is ~29 °C, the cooling mode glazing panel lowers the temperature by about 2 °C, while the heating mode glazing panel permits solar heating and raises the temperature of an undersurface sunlight absorber to ~24 °C above the ambient temperature.

## Application

Although many photonic technologies enhance radiative cooling with performance and esthetics, many of them do not satisfy the market requirements due to material reliability, durability, and robustness. The most significant obstacles in applying radiative cooling in systems is scalable mass manufacturing. Most photonic structures are realized in lab-scale devices, and considerably more complicated photonics are rather challenging to build. However, despite many obstacles, efforts to apply radiative cooling technology, which does not require any energy, to the industry are constantly being studied. In this section, both current radiative cooling applications and those under investigation are reviewed. Some potential application areas are covered, including space cooling of vehicles and buildings, solar cell, energy and dew water harvesting, and textile cooling.

### Vehicle

**Fig. 11:**
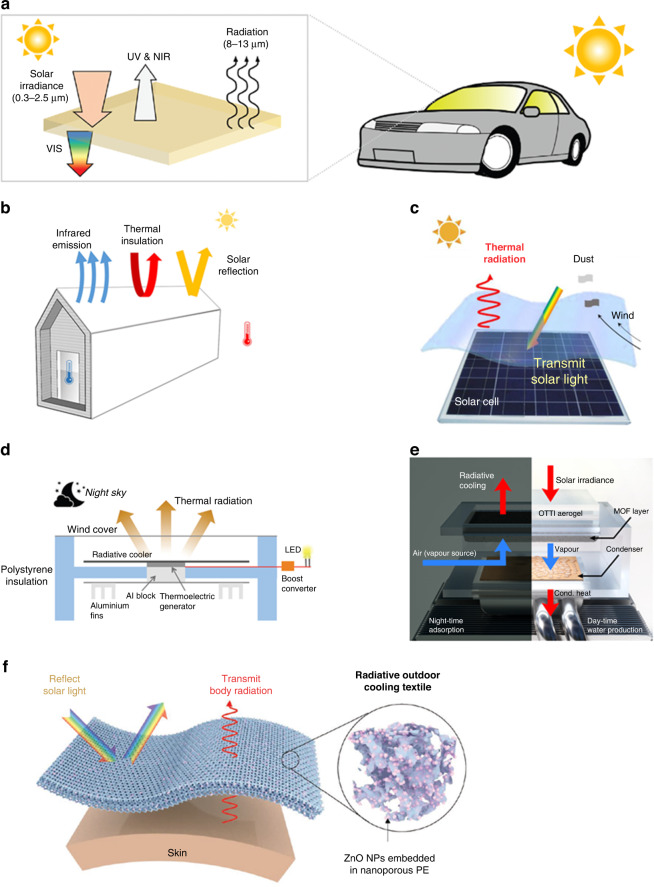
Photonic radiative cooling in applications. **a** Schematic of transparent radiative cooler in the vehicle. Reproduced with permission^166^. Copyright 2021, Wiley-VCH. **b** Schematic of hollow fiber radiative cooling in building. Reproduced with permission^216^. Copyright 2021, ACS. **c** Schematic of transparent radiative cooler and self-cleaning in solar cell. Reproduced with permission^160^. Copyright 2021, Elsevier. **d** Schematic of energy harvesting with radiative cooler and thermoelectric generator in night time. Reproduced with permission^239^. Copyright 2019, Elsevier. **e** Schematic of the water harvesting device with radiative cooler. Reproduced with permission^243^. Copyright 2018, Springer Nature. **f** Schematic of personal temperature regulation using radiative cooling. Reproduced with permission^244^. Copyright 2018, Wiley-VCH

Inner space cooling is still challenging even though radiative cooling has been well investigated. A cooling solution for confined spaces where extremely high temperatures might occur is highly desired. For instance, the greenhouse effect where automobile windows are opaque to outgoing mid infrared thermal radiation causes temperatures of vehicles inside to increase up to 60 °C^211^, which increases the cooling load in the cabin; and puts the occupants, particularly young children, at risk of hyperthermia and heat stroke^212^. Although conventional air conditioning can decrease the temperature, nonrenewable fossil energies must be consumed for cooling, contributing to the decrease in energy efficiency. To improve the cooling energy efficiency of vehicles, therefore, a new technology adopted on the vehicle exterior must be developed. A radiative cooler can be used as a window or roof. Kim et al.^166^ developed a transparent radiative cooler that transmits in the visible range, reflects NIR, and emits thermal radiation to the atmospheric window to reduce the interior temperature throughout the day (Fig. 11a). In an experiment, sunlight transmitted through a transparent object was trapped by an absorbing chamber that resembled a car. The transparent cooler could reduce the interior temperatures by up to 14 °C. Heo et al.^213^ presented opaque radiative cooling on a vehicle roof, describing an emitter that can selectively emit heat into space as a top layer and absorbs thermal radiation from the inside vehicle in a broad range of spectral ranges to dissipate heat to the outside vehicle. The suggested design includes selective emission on the top side that matches the atmospheric windows to reduce the disturbance from atmospheric radiation and broadband emission on the bottom side to ensure broad absorption of internal thermal radiation. An outdoor experimental setup designed to resemble an automobile produced an inside temperature reduction of up to 4 °C.

### Buildings

Buildings consume 40% of the total energy used in heating, ventilation, and air conditioning systems that use passive energy for improving efficiency^214,215^. Integrating radiative cooling technology tremendously improves energy saving and reduces CO_2_ emission reduction. Hence, integrating passive cooling techniques in the building system greatly reduces the energy consumption of cooling systems. For building applications, efforts and studies to implement radiative cooling systems, such passive^113,216–223^ and active cooling^108,195,224–234^, as into the building have been already tried. Here, passive and active systems in radiative cooling are briefly introduced. The paint-format microparticles, which are simple to integrate with structures’ roofs inexpensively, are gaining attention in building cooling applications (Fig. 11b)^216^. The radiative cooling of microparticles of Al_2_O_3_, and SiO_2_ that have large bandgap energy and high IR emission exhibits high solar reflection of 97% in the visible range and thermal emission of 93% in mid-IR. In an active system, air and water are suitable heat exchange mediums, which requires external forces that actively circulate the air and water to boost cooling efficiency and improve temperature control. When active radiative cooling is incorporated in the condenser side of buildings’ cooling systems, the water as medium can be chilled up to 10.6 °C below the surrounding air, and improve energy efficiency^234^.

### Solar cell

Commercial solar cells that convert solar energy directly into electricity have an energy conversion efficiency of up to 20%^235^. Materials with higher bandgap energies than targeting photons energies do not absorb energy and get converted. The widely used material in solar cells, silicone, which absorbs the most visible light in the bandgap of 1.9–3.1 eV^236^, has a bandgap of 1.1 eV. Therefore, most solar irradiation converts the heat and heats the solar cells; the temperature of these cells easily exceeds 60 °C, which gives rise to a decrease in the efficiency of solar cells up to 0.5% for a 1 °C rise in temperature^237^. The main purpose of cooling solar cells is to decrease the operating temperature, unlike for other radiative cooling applications where the aim is to drop the surface temperature^238^. Hence, a radiative cooler that emits heat through the atmospheric window is not necessary, but a broadband emitter is preferred for solar cell cooling due to the cost benefits. Recently, many ways of radiative cooling technologies on solar cells by photonic structure have been demonstrated. In addition, Chen et al.^160^ introduced radiative cooling with a surface cleaning function to improve the efficiency of solar cells (Fig. 11c).

### Energy and water harvesting

Due to the fast-expanding effects of climate change, there is a worldwide effort to find renewable and recyclable energy sources to help reduce carbon emissions. Although photovoltaic (PV) technology has already been established and tested, it cannot be utilized at night, causing an imbalance in power production. A radiative cooling provides potential solution for this problem. Combining a thermoelectric generator (TEG) with a radiative cooling surface can generate power even at night. It is possible to produce a noticeable temperature difference at night by combining TEGs and a radiative cooling. To generate energy at night, Raman et al.^239^ introduced the radiative cooling in the thermoelectric module of the cold side that faces the sky and vents heat into space while simultaneously heating its warm side with the surrounding air (Fig. 11d). This thermoelectric generator with a cooler showed a power generation of 25 mW/m^2^, which is sufficient to power a light emitting diode (LED). Recently, Zhao et al.^240^ improved the performance of radiative cooling driven thermoelectric generator and showed the power density of 291 mW/m^2^ by optimizing its geometry and operating condition.

Dew collection can be the most feasible water harvesting method, especially in an arid area, due to being more cost-effective and less impacted by geographic restrictions than other water harvesting technologies^241^. When the substrate’s temperature is below the dew-point temperature at night, the high humidity causes dew water to collect on the substrate’s surface. The key to collecting dew water at night is the cold condensation surface, which can be reached by a passive radiative cooling method that transfers heat to the outer space. Recently, many water harvest technologies using radiative cooling have been demonstrated after the advent of research on radiative cooling dew condensers in the 1960s^242^. In addition, Kim et al.^243^ introduced air-cooled sorbent water harvesting device combining metal–organic frame, which can capture more water than conventional sorbents (Fig. 11e). This system demonstrated water can be harvested ~0.28 L/kg cycle daily.

### Textile

Traditional fabrics are effective thermal transmitters, but because of their inherent IR opacity, which acts as a radiation shield, radiation from the skin is prevented. To provide thermal comfort, IR transparent fabrics such as polyethylene (PE) is considered for desirable material. Using nanoporous PE fabrics in the environment, considerable improvements in human body cooling have been made^76,241,244–246^. For fabricating commercial fabrics, nanoporous PE fabrics can be created on a large scale^247^. Recently, there have been other attempts to employ radiative cooling for outdoor cooling, which requires textile materials with high reflection in solar irradiation and strong transmission in thermal emission^84,248–255^. Cai et al.^244^ introduced a textile material using radiative cooling combining nanoporous polyethylene with zinc oxide nanoparticles (Fig. 11f). More than 90% of the solar radiation is reflected; however, up to 80% of the heat radiation from the human body is transmissible. By covering a simulated skin with the textile material, we demonstrated that in contrast to cotton, this textile material can prevent the skin from overheating by more than 10 °C in typical outdoor conditions with a peak solar irradiation of over 900 W/m^2^, showing radiative cooling for personal outdoor cooling.

## Summary and perspectives

For decades, many radiative cooling studies have been conducted to overcome the performance limitations using photonic technologies. For example, broadband and selective emissions in mid-IR and high reflectance in the solar spectral range have already been demonstrated. In addition, emission in ultra-high subambient temperature of 60 °C below ambient temperatures^36^, protecting ice under the sun^256^, and combining radiative cooler with evaporative cooler for daytime cooling^257^ have been demonstrated. Even though photonic technology assists in overcoming limitations in radiative cooling, three main obstacles are yet to be overcome for use in practical settings in the radiative cooling field: variability in any environment and preservability of esthetic and compatibility with large-scale production.

The radiative cooling process is significantly influenced by the climate conditions such as humidity, clear or cloudy sky, regional wind speeds, and changing weather conditions at various places^258,259^. Cooling efficiency decreases when the sky is partially or even entirely covered, the radiative cooling effect may still exist, however, the cooling power may be significantly diminished. Hence, these elements must be considered when designing an optimal profile in a radiative cooler. For many years, radiative coolers adaptable to their environment temperatures have been created utilizing thermochromic material with photonic methods. As reviewed in the previous section, VO_2_, the well-known temperature-based phase transition, has been implemented in the smart window film to control thermal emissivity based on ambient temperature, increasing emissivity in the mid-IR in summer and, suppresses the emissivity in the mid-IR in winter. In addition to this film, tunable mid-IR smart paint modulates radiative cooling and solar heating in building. This novel paint comprises specific sized hollow glass beads to reflect the solar irradiation embedded in PDMS to dissipate heat in mid-IR to the outer space, and thermochromic chameleon microcapsules to switch colors when below or above the critical temperature. However, although unidirectional cooling, a weakness of radiative cooler, was overcome, most of them are not thoroughly considered for limited optical characteristics, operating temperatures, or mass production. To reduce solar absorption and enable transitions at lower temperatures, future research should focus on creating advanced photonic techniques with PCMs.

Esthetic preservation on application also remain in hurdles. Many radiative coolers show white or silvery surface, and these coolers interfere with the existing design color when applied to the application. The formation mechanism of color can generally be divided into pigmentary and structural colors. To enhance the cooling effect, instead of using pigment, which produces color by intrinsic absorption of chemical substance, structural color should be implemented by using either retroflective structure that adjusts the size of the sphere according to that of the incident wavelength or multilayer structure. The optical photonic materials and structures for color radiative cooler can be obtained by machine learning^148,260^. Colors other than white have a higher temperature than white, and the temperature increases further if pigments are used for the color. Hence more advanced photonic approaches are investigated to overcome the necessary for coloration.

Radiative cooling technology must be able to be integrated into the current mass manufacturing system to be used in daily life. The manufacturing procedure should be cost-effective and material compatible. From the photonic point of view, the fabrication procedure for 2-D and 3-D photonic radiator structures is challenging, making their commercial application challenging^261,262^. Laser lithography techniques have been explored to fabricate these complex structures^103,263,264^; however, some complicated structures such as biomimetic and pyramidal structures that were reviewed in this article still need to be fabricated. Some scalable photonic coolers such as paint^75,220^, fabric^76,86,253^, and film^70^ have been reported, but their durability, cost-effectiveness, and mechanical strength need to be developed. In addition, although scalable photonic coolers are confined in opacity, scalable photonic radiative transparent cooler needs to be developed.

In summary, the fundamental principles of photonic radiative cooling were reviewed emphasizing recent progress in the field. Photonic nanostructures are crucial for increasing the efficiency. Several nanophotonic structures such as multilayer thin film, photonic crystal, metasurface; derived from nature, and associated design procedures were thoroughly discussed. Progress has also been made in terms of adding functionality. Photonic integration with new functionality significantly enhances the efficiency of radiative cooling technologies. As evidenced in this review, a list of colored, transparent, and switchable radiative cooling applications has been developed. The benefits of radiative cooling can support commercial applications such as reducing cooling loads in vehicles, increasing the power generation of solar cells, generating electricity, saving water, and personal thermal regulation. As the increase in carbon dioxide and global warming effect worsen today, radiative cooling technologies that do not require electricity play an important role in reducing energy consumption, making a substantial contribution to achieving carbon neutrality.

## Data Availability

The data that support this research’s findings are available and can be provided based on the request to the corresponding authors.
